# Supplementary material for: Aspartame and Phe-Containing Degradation Products in Soft Drinks across Europe
Source: Nutrients. 2020 Jun 24;12(6):1887. doi: 10.3390/nu12061887 (PMC7353418; doi:10.3390/nu12061887)
Supplement: Supplementary file 1 [file nutrients-12-01887-s001.pdf]

Supplementary

Table S1. Carry-over experiment for aspartame and its degradation products.

	**APM**	**Phe**	**DKP**	**Asp–Phe**
Mean sample low (L)	226.6	1.9	0.0	24.8
Mean sample high (H)	1882.0	1.7	60.3	136.7
Mean L-L	226.8	1.9	0.0	24.7
Mean L-H	226.0	1.8	0.0	24.7
3*SD L-L	23.80	0.63	0.00	1.21
Difference L-L - L-H	0.75	0.08	0.00	0.05
Carry over	No	*	No	No

Concentrations in µmol/L. APM; Aspartame, Phe; phenylalanine, DKP; diketopiperazine, Asp–Phe; aspartylphenylalanine The mean and standard deviation of sample low after sample low (L-L: L2, L5, L6 en L7) and sample low after sample high (L-H: L3, L4, L8, L9 en L10) were calculated. Carry-over was considered present when mean L-H – mean L-L <3* SD of L-L. * Carry-over of Phe could not be calculated from these samples, as mean sample high was similar to mean sample low.

Table S2. Aspartame, degradation products and total phenylalanine concentrations in 111 soft drinks from across Europe. Similar drinks are grouped and clustered.

		Soft drink	Country	APM (µmol/L)	Phe (µmol/L)	DKP (µmol/L)	Asp-Phe (µmol/L)	Total Phe (µmol/L) Mean (SD)
Group A	A	Fanta Exotic	NL	368	8	4	15	395
		Fanta Exotic	BE	360	6	5	18	389
		Fanta Lemon	BE	0	2	0	0	2
		Fanta Lemon	FI	0	0.8	0	0	0.8
								262 (225)
	B	Fanta Orange	FR	0	13	0	0	13
		Fanta Orange	TR	0	3	0	0	3
		Fanta Orange	DE	0	3	0	0	3
		Fanta Orange	NL	197	10	0.3	13	219
		Fanta Orange	BE	0	7	0	0	7
		Fanta Orange	FI	198	6	12	23	239
								81 (115)
	C	Fanta No Sugar Lemon	BE	594	5	16	29	644
		Fanta No Sugar Orange	BE	1040	8	21	35	1104
		Fanta Zero Orange	NL	950	10	21	38	1019
		Fanta Zero Orange	FI	934	8	53	71	1066
		Fanta Zero Peach Apricot	NL	451	6	3	17	476
		Fanta Zero Sugar Orange	GB	737	9	32	50	827
		Fanta Zero Sugar Orange	ES	255	6	9	18	288
								775 (316)
Group B	A	7-up Free	NL	1570	2	74	45	1691
		7-up free	BE	1315	0.2	48	48	1411
		7-up Free	ES	1145	3	71	53	1272
		7-up Free	FI	1025	2	63	75	1165
								1385 (228)
	B	Sprite	NL	790	1	7	27	826
		Sprite	FI	654	3	56	72	785
		Sprite	FR	394	0	10	25	429
		Sprite	GB	349	0	21	41	411
		Sprite	TR	362	0	17	23	402
		Sprite	BE	0	0	0	0	0
		Sprite	DE	0	0	0	0	0
								408 (329)

	C	Sprite (low in sugar)	ES	351	0.01	22	28	401
		Sprite Lemon Lime & Cactus	NL	676	2	15	33	726
		Sprite Lemon Lime & Cranberry	NL	716	2	19	34	770
		Sprite No Sugar	BE	786	0	21	37	843
		Sprite No Sugar	DK	773	0	27	43	842
								716 (183)
Group C	A	Coca Cola	TR	0	0	0	0	0
		Coca Cola	FI	0	0	0	0	0
								0 (0)
	B	Coca Cola Light	BE	792	0	20	47	858
		Coca Cola Light	NL	740	2	14	50	805
		Coca Cola Light	SE	726	0	19	54	799
		Coca Cola Light	DE	235	3	20	90	348
		Coca Cola Light Exotic Mango	SE	630	2	39	81	750
		Coca Cola Light Exotic Mango	DE	506	7	45	129	687
		Coca Cola Light Exotic Mango	NL	502	6	28	136	672
		Coca Cola Light Ginger Lime	NL	630	4	21	115	770
		Coca Cola Light Ginger Lime	DE	267	3	22	76	368
								673 (188)
	C	Coca Cola Zero Sugar	BE	1255	0.04	40	63	1359
		Coca Cola Zero Sugar	FR	1275	0	28	47	1350
		Coca Cola Zero Sugar	DK	1180	0.7	30	104	1314
		Coca Cola Zero Sugar	GB	1085	4	71	140	1300
		Coca Cola Zero Sugar	FI	1018	4	63	102	1186
		Coca Cola Zero Sugar	NL	1074	2	23	59	1157
		Coca Cola Zero Sugar	ES	389	0	10	22	422
		Coca Cola Zero Sugar	DE	151	12	47	100	310
		Coca Cola Zero Sugar	TR	0	0	0	0	0
		Coca Cola Zero Sugar Lemon	BE	739	0.2	29	53	821
		Coca-Cola Zero Sugar Cherry	NL	730	0.9	7	27	766
		Coca-Cola Zero Sugar Lemon	NL	786	0.8	8	28	823
		Coca-Cola Zero Sugar Vanilla	NL	770	1	10	32	813
								894 (439)
	D	Pepsi Max Cherry No Sugar	FI	1310	26	184	331	1850
		Pepsi Max Lime No Sugar	FI	1480	15	145	228	1867
		Pepsi Max No Sugar	GB	1640	7	111	198	1955
		Pepsi Max No Sugar	SE	1410	21	156	318	1905
		Pepsi Max No Sugar	FI	1530	9	114	203	1856

								1887 (44)
	E	Pepsi Max Zero Caffeine Zero Sugar	BE	1500	13	112	222	1847
		Pepsi Max Zero Sugar	DE	1790	3	64	156	2013
		Pepsi Max Zero Sugar	BE	1670	4	64	159	1897
		Pepsi Max Zero Sugar	NL	1635	7	86	164	1892
		Pepsi Max Zero Sugar Cherry	BE	1270	30	166	319	1784
		Pepsi Max Zero Sugar Cherry Flavour	NL	1760	6	65	161	1991
		Pepsi Max Zero Sugar Ginger Flavour	NL	1580	12	107	221	1920
		Pepsi Max Zero Sugar Lemon	BE	1260	2	46	80	1388
		Pepsi Max Zero Sugar Lemon Flavour	NL	1180	7	68	114	1369
								1789 (243)
Group D	A	Aquarius Zero Sugar Orange	ES	130	0	4	15	148
		Bullit Sugar Free	NL	557	2	29	30	618
		Bullit Sugar Free Citrus Splash	NL	385	5	45	52	486
		Bullit Sugarfree Full Berry	NL	363	6	48	52	470
		Diet Pepsi	GB	999	3	68	96	1166
		Dr Pepper	GB	356	0	18	47	420
		Dr Pepper	FI	0	0	0	0	0
		Freeway Cola Zero	FI	280	5	40	93	418
		Freeway Cola Zero Sugar	NL	405	1	2	26	434
		Golden Power Energy Drink met Taurine Zero	NL	504	2	26	34	565
		Hero Cassis Zero	NL	247	3	1	16	267
		Highway Cassis	NL	451	4	20	39	514
		Highway Cola	NL	101	2	0.2	33	136
		Highway Cola Light	NL	596	14	74	141	825
		Highway Lemon Lime	NL	116	0.9	5	16	137
		Highway Orange	NL	226	2	16	26	271
		Irn-Bru	GB	412	0	24	45	481
		Jaffa Ananas Sokeriton	FI	1085	10	109	128	1332
		Jaffa Appelsiini	FI	0	1	0	0	1
		Jaffa Appelsiini	FI	0	1	0	0	1
		Jaffa Appelsiini Sokeriton	FI	1340	4	49	61	1455
		Jaffa Lime-Verigreippi Sokeriton	FI	302	2	18	27	349
		Jaffa Vesimeloni-Vadelma Sokeriton	FI	725	0	23	37	784

Jumbo Cola Coconut Flavour 0% Sugar	NL	667	4	34	57	762
Jumbo Cola Light	NL	405	24	94	146	667
Jumbo Cola Zero Sugar	NL	119	2	31	51	1269
Jumbo Cola Zero Sugar Cherry Taste	NL	456	6	32	77	570
K Menu Cola Light	FI	1525	12	123	272	1931
Look Cola Zero	FR	1630	3	60	119	1813
Lucozade Energy	GB	286	0	12	24	322
Mountain Dew	FI	0	0	0	0	0
Nestea No Sugar Lemon	ES	173	0	6	15	194
Pepsi	FI	0	0	0	0	0
Red Bull Sugarfree	NL	552	3	40	46	641
Red Bull Sugarfree	ES	549	1	48	36	634
Red Bull Zero Callories	NL	451	2	21	29	503
Redbull Zero Sugar	SE	423	0.1	32	42	497
Ribena Light Blackcurrant	GB	734	0.5	38	55	827
River Cola Zero	NL	1020	5	49	113	1187
Royal Club Tonic with a Hint of Grapefruit	NL	103	1	1	14	119
						580 (484)

APM; aspartame, Phe; phenylalanine, DKP; diketopiperazine, Asp-phe; aspartylphenylalanine, NL; Netherlands, BE; Belgium, FI; Finland, FR; France, TR; Turkey, DE; Germany, GB; England, ES; Spain, DK; Denmark, SE; Sweden.
